# A Behavioral Comparison of Male and Female Adults with High Functioning Autism Spectrum Conditions

**DOI:** 10.1371/journal.pone.0020835

**Published:** 2011-06-13

**Authors:** Meng-Chuan Lai, Michael V. Lombardo, Greg Pasco, Amber N. V. Ruigrok, Sally J. Wheelwright, Susan A. Sadek, Bhismadev Chakrabarti, MRC AIMS Consortium, Simon Baron-Cohen

**Affiliations:** 1 Department of Psychiatry, Autism Research Centre, University of Cambridge, Cambridge, United Kingdom; 2 School of Psychology and Clinical Language Sciences, Centre for Integrative Neuroscience and Neurodynamics, University of Reading, Reading, United Kingdom; The University of Queensland, Australia

## Abstract

Autism spectrum conditions (ASC) affect more males than females in the general population. However, within ASC it is unclear if there are phenotypic sex differences. Testing for similarities and differences between the sexes is important not only for clinical assessment but also has implications for theories of typical sex differences and of autism. Using cognitive and behavioral measures, we investigated similarities and differences between the sexes in age- and IQ-matched adults with ASC (high-functioning autism or Asperger syndrome). Of the 83 (45 males and 38 females) participants, 62 (33 males and 29 females) met Autism Diagnostic Interview-Revised (ADI-R) cut-off criteria for autism in childhood and were included in all subsequent analyses. The severity of childhood core autism symptoms did not differ between the sexes. Males and females also did not differ in self-reported empathy, systemizing, anxiety, depression, and obsessive-compulsive traits/symptoms or mentalizing performance. However, adult females with ASC showed more lifetime sensory symptoms (*p* = 0.036), fewer current socio-communication difficulties (*p* = 0.001), and more self-reported autistic traits (*p* = 0.012) than males. In addition, females with ASC who also had developmental language delay had lower current performance IQ than those without developmental language delay (*p*<0.001), a pattern not seen in males. The absence of typical sex differences in empathizing-systemizing profiles within the autism spectrum confirms a prediction from the extreme male brain theory. Behavioral sex differences within ASC may also reflect different developmental mechanisms between males and females with ASC. We discuss the importance of the superficially better socio-communication ability in adult females with ASC in terms of why females with ASC may more often go under-recognized, and receive their diagnosis later, than males.

## Introduction

Autism spectrum conditions (ASC) are neurodevelopmental and are diagnosed on the basis of difficulties in social interaction and communication, alongside the presence of restricted interests, difficulties adapting to change, and repetitive, stereotyped behavior [1], [2]. ASC is one of the most common neurodevelopmental conditions, affecting approximately 0.6 to 1.57% of the general population [3], [4], [5]. Within ASC, males outnumber females with a sex ratio of 4.3∶1 [6]. This asymmetry in sex ratio has been known for many decades [7], and raises an important, unanswered question: *Are there sex differences in autism?* Although seemingly straightforward, it is not simple to answer. This question needs to be addressed at three different levels: *prevalence*, *neurobiological/developmental mechanism, and behavior*. In this paper we report an experiment that addresses this question at the behavioral level.

### Sex differences in prevalence in ASC

The initial description of children with “autistic disturbances of affective contact” by Leo Kanner described 8 boys and 3 girls [8]. Similarly, the report on “autistic psychopathy” by Hans Asperger concerned 4 boys and no girls [9]. Although these were small clinic samples, this male bias was also seen in the early epidemiological studies of classic autism with concurrent intellectual disability, where the male∶female ratio was 3–4∶1 [7], [10], [11], [12], [13]. Among those with low IQ, the sex ratio decreased to 2∶1 [10], [14] but was nevertheless still present. Despite better recognition of ASC today, these sex ratios and their relation to intellectual ability are consistent with those reported 30 years ago. The sex ratio for individuals with average intelligence is 5.5∶1, but 1.95∶1 in those with intellectual disability [6]. However, these studies may have underestimated the number of females with ASC if they have a “non-male-typical” presentation, and if females with undiagnosed ASC make more effort to camouflage their difficulties [15], [16], [17], [18], [19]. Thus, studies comparing the behavior of males and females with ASC are still needed.

### Sex differences and neurobiological/developmental mechanism

The male-bias in ASC has also raised the question of how the sex ratio might be relevant to the etiological and/or developmental mechanisms underlying ASC. There are at least four complementary views that are not necessarily mutually exclusive. First, it may be that *different mechanisms* are involved in ASC for males and females. Evidence in support of this includes sex differences in developmental cognitive profile [20] or underlying biology [21]. Second, it may be that females are less *vulnerable* to developing ASC because of innately protective mechanisms [10], [22], [23], [24], [25]. This fits with the finding that various types of early onset neurodevelopmental conditions affect males more than females [26]. Evidence in support of this view would need to show that, given the same level of autistic symptom severity, females show greater neurobiological changes than males compared to their neurotypical counterparts [27]. Third, it may be that males and females are equally at risk for ASC (in terms of genetic predisposition), but other factors enable females to better *compensate* for these risks [28]. Evidence supporting this view would need to show, at the cognitive and/or neurobiological levels, what these factors are and how they compensate for the vulnerability throughout the lifespan.

Finally, it may be that at the cognitive and/or biological levels, ASC is *an extreme of the male brain* (EMB) in the domains of empathy and systemizing [9], [29], [30]. The prediction from this hypothesis is that sexual dimorphism in empathy and systemizing within the typical population is reduced or absent in ASC, and that ASC involves a “hyper-masculinized” cognitive style (and probably also in the underlying biology of this cognitive profile). The EMB theory predicts that females with ASC will be cognitively (and even biologically) more “male-like”, and this could mean they are either (i) comparable to males with ASC, (ii) intermediate between typical males and males with ASC, or (iii) comparable to typical males. Results from self-report questionnaire studies support prediction (i) since typical sex differences in autistic traits, empathy and systemizing in adults are absent in ASC [31], [32]. This evidence extends to parent-reported autistic characteristics in childhood [33] and in adolescence [34]. Similarly, no sex differences within ASC are found on the child versions of the Empathy Quotient and Systemizing Quotient [35], the Childhood Autism Spectrum Test [36], and the Quantitative Checklist for Autism in Toddlers [37]. Given that in the general population males score higher than females on all of these instruments, the *absence* of a sex difference in autism is consistent with the view that females with ASC show a masculinized profile. These theoretical viewpoints may not be mutually exclusive, and identifying if they overlap will be important in future research.

### Sex differences in behavior in ASC

Current international criteria for diagnosing ASC are based on behavior [2], [38], [39]. If males and females with ASC show different behavioral phenotypes [15], [16], [17], [18], [19], [28], we may need sex-specific behavioral or cognitive criteria for defining ASC, in addition to or replacing the current criteria.

When studying sex differences in ASC there is a need for close matching on age and IQ. Early studies used community or clinical samples and were not always successful in matching participants. Thus, some of the highlighted behavioral sex differences, such as greater unusual visual responses and motor stereotypy and less appropriate play in boys [40], [41], and more appropriate interests [17], [18], [42] and better superficial social and communication skills in girls [15], [16], [18] may have been confounded by factors such as age or intellectual level.

Studies that did match the groups are inconsistent. McLennan et al. [43] tested 21 boys and 21 girls (aged 6–36 years old) without marked intellectual disability (IQ>60). Boys had more severe autistic symptoms in early social communication development, measured by the Autism Diagnostic Interview [44]. In another example, Carter et al. [20] found that 68 male and 22 female toddlers with ASC (aged 1.7–2.8 years old) had different cognitive and developmental profiles. Girls had better visual reception and boys had better motor and communication skills. Finally, Hartley et al. [45] tested 157 boys and 42 girls with ASC (aged 1.5–3.9 years old) using the Autism Diagnostic Observation Schedule (ADOS) [46] and found that girls were more impaired on the communication domain, whereas boys showed more restricted/repetitive/stereotyped interests and behaviors. Girls also had more concurrent anxious/depressed symptoms and sleep problems.

In contrast, other studies using matched samples report no differences between males and females with ASC. Tsai et al. [47] found that 19 boys and 19 girls (mean age 6 years old) with classical autism were equally impaired in their cognitive, physical and self-help abilities. Pilowsky et al. [48] also found no sex differences on the Autism Diagnostic Interview-Revised (ADI-R) [49] and the Childhood Autism Rating Scale (CARS) [50] between 18 boys and 18 girls with ASC (aged 3–30 years old) who had intellectual disability. Holtmann et al. [51] matched 23 male and 23 female children and adolescents with ASC (aged 5–20.2 years old) without intellectual disability (IQ>70, mean score 88.8) and found no differences in autistic presentation. However, females showed more parent-reported coexisting psychopathology, particularly social, attention, and thought problems. Lastly, several questionnaire-based studies have found no evidence of behavioral sex differences in ASC [31], [33], [34], [35], [36], [37].

The similarities and differences between males and females with ASC may be indicative of the marked heterogeneity of ASC, and indicates the need to consider sub-groups stratified by age, IQ, and autistic symptom severity. The demographic background of the sample population as well as the recruitment strategies may also affect the outcomes of comparison.

### Behavioral sex differences in adults with ASC

The above studies all focus on children or mixed-age samples. To our knowledge there are no studies addressing behavioral sex differences in *high-functioning adults with ASC*, apart from questionnaire-based studies. This is striking given the increasing awareness of the need to improve assessment, diagnosis and services for adults on the autistic spectrum [27], [52], and given that women on the spectrum are often recognized later than males, and may be misdiagnosed [15], [16], [53], [54], [55]. To fill these gaps we conducted a study to test IQ- and age-matched adult males and females with ASC using a large battery of behavioral and cognitive measures. Our intent is to extend prior questionnaire-based studies in adults to a broader range of measures in the clinical domain as well as performance-based measures of cognitive abilities.

## Materials and Methods

### Ethics statement

Informed written consent was obtained for all participants in accord with procedures approved by the Suffolk Research Ethics Committee.

### Participants

Participants were recruited through the UK Medical Research Council Autism Imaging Multicentre Study (MRC AIMS) consortium. Recruitment was conducted through advertisements sent to national and local autism support organizations and support groups in England and Wales, referral from diagnostic clinics for adults with autism or Asperger syndrome, and via the participant database of the Autism Research Centre, University of Cambridge (http://www.autismresearchcentre.com). The same inclusion criteria were applied to both male and female groups: aged between 18 to 45 years, with English as first language, without intellectual disability (IQ≥70), and having a formal clinical diagnosis of autistic disorder or Asperger syndrome, based on DSM-IV [2] or ICD-10 [39] criteria, from a chartered psychiatrist or clinical psychologist working in the UK National Health Service. Exclusion criteria for both groups included a diagnosis of current or historical psychotic disorders, substance-use disorders, medical conditions associated with autism (e.g. tuberous sclerosis, fragile×syndrome), intellectual disability, epilepsy, hyperkinetic disorder, and Tourette's syndrome. Under these criteria, 83 ASC participants (45 males and 38 females) took part in a series of behavioral and cognitive assessments at the Autism Research Centre, University of Cambridge.

### Behavioral assessments

#### Subject characteristics

The main childhood caregiver of each participant was interviewed using the Autism Diagnostic Interview-Revised (ADI-R) [49]. The ADI-R is a standardized, semi-structured interview schedule based on the DSM-IV and ICD-10 diagnostic concepts of autism, exploring an individual's early development, acquisition and/or loss of language skills, language and communication functioning, social development and play, interests and behavior, general behavior and caregiver concerns via 93 subject items. Information used for diagnosis was based on the caregiver's report of the individual's developmental history and behavior across time and place. On average the interview lasted 2.5 to 3.5 hours. Caregiver's descriptions of the individual's childhood (or “ever”) and current behaviors were coded immediately during the interview, relying on the interviewer's judgment of the detailed descriptions of behaviors that correspond to developmental deviance. In the present study, the “diagnostic algorithm” scores were used for analysis, as most studies do, which reflect three areas of functioning: Reciprocal Social Interaction, Communication and Language, and Repetitive, Restrictive and Stereotyped Behavior (RSB). Individuals who reached the cut-off in all the three domains, plus an onset of symptom before age of 36 months are given an ADI-R classification of “autism”.

Beside the three diagnostic algorithm domain scores, for the purpose of investigating the sensory aspect, we created an “unusual sensory response” composite score from three ADI-R items that specifically addressed sensory behaviors, namely item 71 “unusual sensory interests”, item 72 “undue general sensitivity to noise”, and item 73 “abnormal, idiosyncratic, negative response to specific sensory stimuli”. This composite score is the sum of the raw “ever” (i.e., lifetime) scores of the three items (raw coding of “9 = N/K or not asked” was coded as 0), giving a range of 0 to 9. Note that only item 71 contributed to the diagnostic algorithm scores (for the RSB domain). Moreover, “history of language delay” was defined as either present or absent for each individual by item 9 “age of first single words” and item 10 “age of first phrases”. Individuals delayed on either or both items were defined as having a history of language delay.

All individuals with ASC were also assessed using module 4 of the Autism Diagnostic Observation Schedule (ADOS) [46]. The ADOS is a standardized activity and interview based semi-structured assessment for current autistic behavioral presentation. Depending on the person's expressive language level, the interviewer can administer one of the four modules. Since our participants were adults with fluent speech, module 4, consisting of 15 activities, was chosen for all participants. On average testing took 45 minutes to an hour. Behaviors of the participant during the session were recorded and coded immediately afterwards into 31 subject items, of which 16 were entered into the “diagnostic algorithm” to describe behavior during natural interpersonal contact in the domains of Social Interaction, Communication, Imagination/Creativity and Stereotyped Behaviors and Restricted Interests. According to the coding algorithm, scores in the domains of Social Interaction, Communication, and the sum of these two contribute to the ADOS classification of “autism”, “autism spectrum”, and “non-autism”. These summary scores were used for analysis, as most studies do. The ADOS has good to excellent psychometric properties, and satisfactory ability to differentiate individuals with and without ASC [46].

For intellectual ability, all participants were assessed by the Wechsler Abbreviated Scale of Intelligence (WASI) [56] that provides measures of verbal, performance, and full-scale IQ.

Participants in both groups also completed three self-report questionnaires measuring their aspects of cognitive style, preferences and traits. The Autism Spectrum Quotient (AQ) [31] is a 50-item questionnaire measuring autistic traits in social skills, attention switching, attention to detail, communication, and imagination. The Empathy Quotient (EQ) [57] is a 40-item questionnaire measuring thought and behavioral characteristics in both the affective and cognitive aspects of empathy. The Systemizing Quotient revised version (SQ) [32] is a 75-item questionnaire measuring the cognitive and behavioral features of “systemizing”, the drive to analyze, understand, predict, control and construct rule-based systems.

Finally, the “Reading the Mind in the Eyes” test (Eyes Test) [58] was completed by each participant. The Eyes Test, composed of 36 items, is an advanced mentalizing task requiring the individual to infer mental status solely from the information in photographs of a person's eyes and the immediate surrounding areas. The AQ, EQ, SQ and Eyes Test have all been shown to have excellent psychometric properties [31], [32], [57], [58]. In addition, there are two important features of these tasks: (i) compared to typical individuals, people with ASC score significantly higher on the AQ, lower on the EQ, higher on the SQ and lower on the Eyes Test; and (ii) typical males, on average, score significantly higher on the AQ, lower on the EQ, higher on the SQ and lower on the Eyes Test compared to typical females.

### Co-occurring psychiatric symptoms

Co-occurring psychiatric symptoms are not uncommon in adults with ASC [59], particularly depression and anxiety. Symptoms of anxiety and depression are also more common in females in the typical population [60]. Obsessive and compulsive traits are phenomenologically related to the RSB domain of ASC and are commonly present conjointly [61], [62]. Each participant therefore filled out three well-validated, commonly used clinical and research instruments: for anxiety the 21-item Beck Anxiety Inventory (BAI) [63], for depression the 21-item Beck Depression Inventory (BDI) [64], and for obsessions and compulsive behaviors the 18-item Obsessive Compulsive Inventory-Revised (OCI-R) [65].

### Statistical analysis

Independent samples t-tests were conducted to examine matching of the male and female ASC groups for age and IQ. Three separate multivariate analysis of (co)variance (MANOVA or MANCOVA) were conducted to examine childhood autistic symptoms (ADI-R algorithm domain scores), cognitive style (AQ, EQ, SQ, and Eyes Test), and co-morbid psychopathology (BAI, BDI, and OCI-R), respectively, in order to take into account the possible inter-dependency among the dependent variables in each cluster. Owing to the highly skewed distribution of the ADOS diagnostic algorithm scores and the ADI-R “unusual sensory response” composite score, nonparametric Mann-Whitney tests were used for these variables. Chi-square test was performed to examine the relationship between sex and history of language delay. A two-way analysis of variance (ANOVA) was then performed to examine the main effects and interaction effect of sex and history of language delay on verbal and performance IQ, respectively. All statistical analyses were performed with the PASW Statistics version 18 (SPSS Inc., Chicago, IL, USA).

## Results

### Participant characteristics

To ensure a non-biased comparison of behavior, male and female adults are best defined as having ASC in childhood by the same behavioral criteria. To be conservative, only individuals who reached ADI-R diagnostic algorithm cut-offs in the three domains of impaired reciprocal social interaction, communication, and repetitive, restrictive and stereotyped behavior (RSB) were included in the following analyses. However, failure to reach cut-off in one of the domains by one point was permitted, to allow for the possible underestimation of early developmentally atypical behaviors in the recall by caregivers whose children are now adults over the age of 18. This criterion resulted in the selection of 62 (33 males, 29 females) out of the total 83 ASC participants (45 males and 38 females) who already had a clinical diagnosis of Asperger syndrome or autistic disorder. These supra-threshold participants all scored above the cut-offs for the domains of impaired reciprocal social interaction and impaired communication, whereas 3 males (9.1%) and 6 females (20.7%) scored one point below in the RSB domain yet scored high on the other two.

The two groups were well matched on chronological age, verbal IQ, performance IQ, and full-scale IQ (Table 1). They were mainly young adults with average or above-average intelligence, and with similar levels of verbal and performance IQ.

**Table 1:** Age and IQ-matched sample.

	Male (N = 33)	Female (N = 29)	Statistics	
	Mean (SD)	Mean (SD)	*t*	*p*
Age (year)	27.0 (7.1)	26.9 (6.7)	0.085	0.933
Verbal IQ	111.5 (15.3)	113.1 (15.4)	−0.413	0.681
Performance IQ	111.1 (16.4)	109.5 (17.5)	0.373	0.711
Full IQ	112.6 (16.3)	112.8 (15.7)	−0.069	0.945

SD: standard deviation.

### Childhood autistic symptoms

The first MANOVA treated sex as the only factor in the model with two levels (i.e., male and female), and the three ADI-R diagnostic algorithm domain scores as the dependent variables. Overall, male and female adults with ASC were not significantly different from each other on childhood ADI-R scores (Wilk's lambda *Λ* = 0.914, *F*_(3,58)_ = 1.826, *p* = 0.153). Separate univariate ANOVAs showed no significant sex differences on the reciprocal social interaction (*F*_(1,60)_ = 0.868, *p* = 0.355), communication (*F*_(1,60)_ = 2.657, *p* = 0.108), and RSB domains (*F*_(1,60)_ = 4.076, *p* = 0.048) after Bonferroni correction for multiple comparisons (Table 2). Chronological age was not correlated with any of these domain scores.

**Table 2:** Comparison of childhood ADI-R algorithm scores by MANOVA and current ADOS module 4 algorithm scores by Mann-Whitney tests.

	Male (N = 33)	Female (N = 29)	Statistics		ES
	Mean (SD) *[range]*	Mean (SD) *[range]*	*F*	*p*	*d*
ADI-R					
Social interaction	18.0 (5.0) *[10–27]*	16.9 (4.8) *[11–29]*	0.868	0.355	0.22
Communication	15.2 (3.5) *[8–22]*	13.6 (4.4) *[8–25]*	2.657	0.108	0.41
RSB	5.7 (2.5) *[2–10]*	4.5 (2.0) *[2–10]*	4.076	0.048	0.53

	Median *[range]*	Median *[range]*	*U (z)*	*p*	*r*
ADOS module 4					
Social interaction	5 *[1–12]*	3 *[0–13]*	308 *(2.425)*	0.015	0.31
Communication	3 *[0–6]*	1 *[0–6]*	215 *(3.778)*	<0.001	0.48
S+C	7 *[1–17]*	4 *[0–19]*	251.5 *(3.215)*	0.001	0.41
RSB	1 *[0–4]*	0 *[0–1]*	236.5 *(3.931)*	<0.001	0.50

ADI-R: Autism Diagnostic Interview-Revised; RSB: repetitive, restrictive and stereotyped behavior; ADOS: Autism Diagnostic Observation Schedule; S+C: ADOS “social interaction+communication” total scores; SD: standard deviation; ES: effect size; *d*: Cohen's *d*; *r*: Pearson *r* (small effect size, *r* = 0.10–0.23; medium, *r* = 0.24–0.36; large, *r*≥0.37).

A separate Mann-Whitney test showed that females displayed significantly higher scores on “unusual sensory response” than males, with medium effect size (female median = 3, mean = 3.1, standard deviation SD = 1.6; male median = 2, mean = 2.3, SD = 1.6; *U* = 321, *z* = 2.097, *p* = 0.036, Pearson *r* = 0.27).

### Current interactive behaviors

Using ADOS module 4 cut-off scores to assess current symptoms, we found that 19 out of the 33 males (57.6%) and 6 out of the 29 females (20.7%) were classified as “autism spectrum” (i.e., Social Interaction+Communication scores≥7); among them, 12 males (36.4%) and 4 females (13.8%) were further classified as “autism” (i.e., Social Interaction+Communication scores≥10). Nonparametric Mann-Whitney tests showed that during immediate interpersonal interaction, female adults with ASC showed significantly less autistic behavior than males in both the socio-communication (*U* = 251.5, *z* = 3.215, *p* = 0.001, *r* = 0.41) and RSB domains (*U* = 236.5, *z* = 3.931, *p*<0.001, *r* = 0.50) with large effect sizes (Table 2). Chronological age did not correlate with any of these symptom scores.

### Cognitive characteristics

A MANCOVA treated sex as the independent variable and the four measures of cognitive characteristics (AQ, EQ, SQ, Eyes Test) as the dependent variables; full-scale IQ was included as a covariate to remove variance in the data due to differences in cognitive abilities which might relate to these measures (Hoekstra, Happé, & Ronald, 2010, conference paper presented at the BPS Developmental Psychology Section Conference, London). Overall male and female adults with ASC differed slightly in their cognitive characteristics (Wilk's lambda *Λ* = 0.841, *F*_(4,56)_ = 2.648, *p* = 0.043). Separate univariate ANCOVAs showed that this significant difference was mainly driven by the females' reporting higher AQ, with a medium effect size (*F*_(1,59)_ = 6.781, *p* = 0.012, Cohen's *d* = 0.65) after Bonferroni correction for multiple comparisons, whereas males and females showed comparable EQ (*F*_(1,59)_ = 0.233, *p* = 0.631), SQ (*F*_(1,59)_ = 0.856, *p* = 0.359), and mentalizing ability on the Eyes Test (*F*_(1,59)_ = 0.046, *p* = 0.832) (Table 3). Chronological age was not correlated with any of these scores.

**Table 3:** Comparison of cognitive profiles by MANCOVA.

	Male (N = 33)	Female (N = 29)	Statistics		ES
	Mean (SD)	Mean (SD)	*F*	*p*	*d*
Self-reports					
AQ	32.8 (7.8)	37.6 (6.8)	6.781	0.012	0.65
EQ	20.1 (10.9)	18.9 (7.6)	0.233	0.631	0.13
SQ	66.9 (23.6)	72.5 (29.2)	0.856	0.359	0.21
Cognitive task					
Eyes Test	22.3 (5.8)	22.7 (6.6)	0.046	0.832	0.06

AQ: Autism Spectrum Quotient; EQ: Empathy Quotient; SQ: Systemizing Quotient Revised version; Eyes Test: correct score on the Reading the Mind in the Eyes test.

### Co-occurring psychiatric symptoms

A significant proportion of adults with ASC showed clinically significant anxiety, depression, or obsessive-compulsive symptoms (Table 4). A final MANOVA treated sex as the independent variable and the three measures of co-occurring psychiatric symptoms as the dependent variables. Overall male and female adults with ASC were not different on these symptoms (Wilk's lambda *Λ* = 0.945, *F*_(3,58)_ = 1.127, *p* = 0.346). Univariate ANOVAs showed no group differences on anxiety, depression or obsessive-compulsive symptoms (Table 5). Chronological age was not correlated with any of these symptom scores.

**Table 4:** Severity distribution of significant co-occurring clinical symptoms.

	Male (N = 33)	Female (N = 29)
	N (%)	N (%)
BAI: clinically significant (score≥8)	21 (63.6%)	21 (72.4%)
Mild anxiety (8–15)	8 (24.2%)	7 (24.1%)
Moderate anxiety (16–25)	11 (33.3%)	9 (31%)
Severe anxiety (26–63)	2 (6%)	5 (17.2%)
BDI: clinically significant (score≥10)	18 (54.5%)	20 (69%)
Mild depression (10–18)	11 (33.3%)	10 (34.5%)
Moderate depression (19–29)	4 (12.1%)	8 (27.6%)
Severe depression (30–63)	3 (9.1%)	2 (6.9%)
OCI-R: compatible to OCD severity (score≥21)	24 (72.7%)	20 (69%)

BAI: Beck Anxiety Inventory; BDI: Beck Depression Inventory; OCI-R: Obsessive-Compulsive Inventory-Revised; OCD: obsessive-compulsive disorder.

**Table 5:** Comparison of co-occurring clinical symptoms by MANOVA.

	Male (N = 33)	Female (N = 29)	Statistics		ES
	Mean (SD)	Mean (SD)	*F*	*p*	*d*
Self-reports					
BAI	13.2 (9.9)	16.1 (10.7)	1.218	0.274	0.28
BDI	13.5 (10.4)	15.5 (8.8)	0.663	0.419	0.21
OCI-R	28.0 (12.6)	25.2 (12.3)	0.790	0.378	0.22

### Sex difference and history of language delay

**Figure 1:**
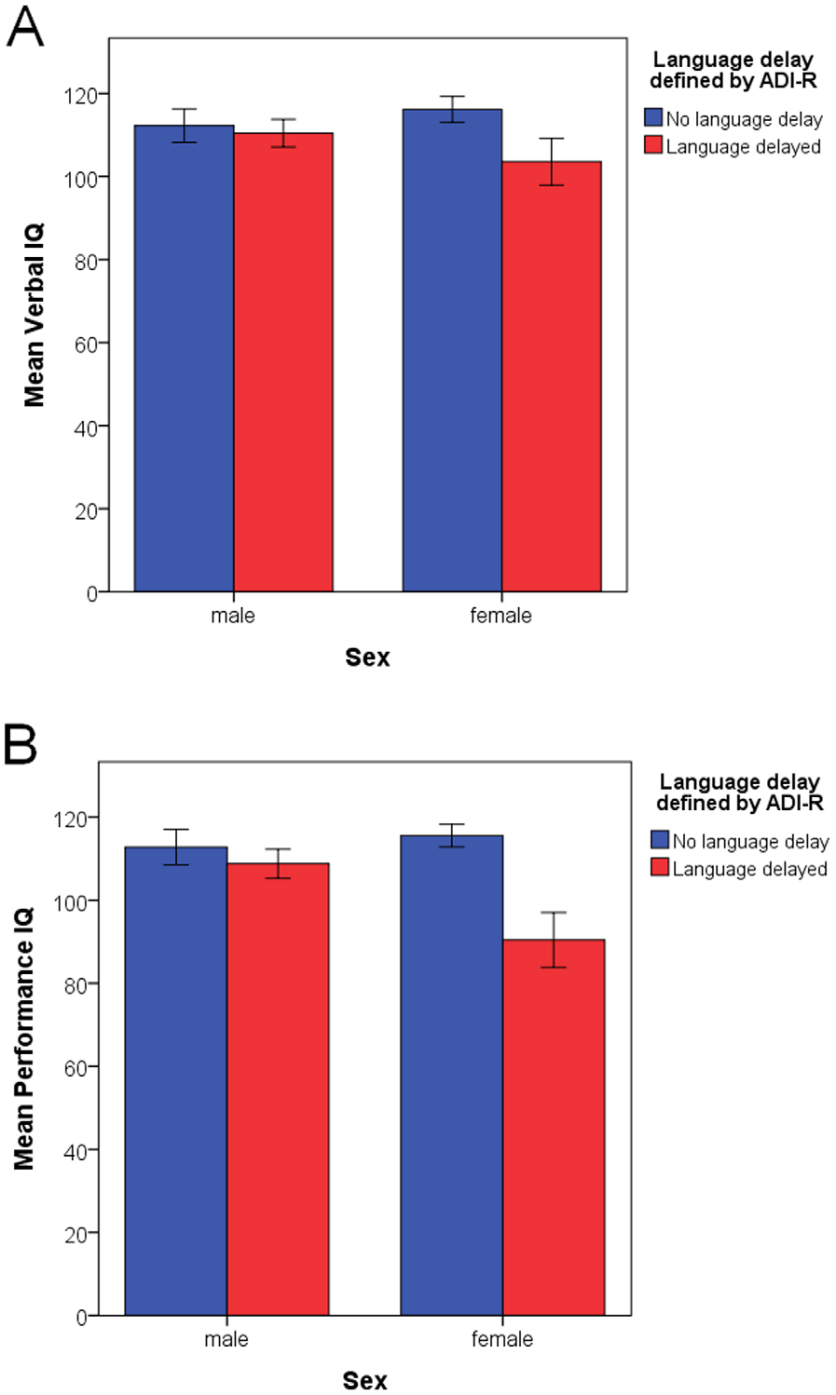
Differential effects of history of language delay on current IQ in male and female adults with ASC. Within adult females with ASC, those with a history of language delay showed marginally lower current verbal IQ (Panel A, right bars, p = 0.053) and significantly lower current performance IQ (panel B, right bars, p<0.001) than those without. This pattern of difference did not exist in adult males with ASC (panel A and B, left bars). Error bar represents standard error of the mean.

There was no association between sex and history of language delay (*χ^2^* = 2.304, contingency coefficient = 0.19, exact significance *p* = 0.18). Two-way ANOVA showed that for verbal IQ, there was no main effect of sex (*F*_(1,58)_ = 0.124, *p* = 0.726) or of history of language delay (*F*_(1,58)_ = 2.888, *p* = 0.095), or any interaction effect (*F*_(1,58)_ = 1.604, *p* = 0.210). For performance IQ, there was no main effect of sex (*F*_(1,58)_ = 3.289, *p* = 0.075), but a significant main effect of history of language delay (*F*_(1,58)_ = 11.459, *p* = 0.001), and a significant interaction effect between sex and the history of language delay (*F*_(1,58)_ = 6.024, *p* = 0.017). Teasing apart the interaction effect by examining each sex separately, within males with ASC we found no difference on performance IQ (*t*_(31)_ = 0.687, *p* = 0.497) between those with a history of language delay (N = 14, mean = 108.8, SD = 13.0) and those without (N = 19, mean = 112.8, SD = 18.7). However, there was a large effect size for a difference in performance IQ (*t*_(27)_ = 4.146, *p*<0.001, Cohen's *d* = 1.80) between females with a history of language delay (N = 7, mean = 90.4, SD = 17.5) and those without (N = 22, mean = 115.6, SD = 12.8) (Figure 1).

## Discussion

This is the first study comparing cognition and behavior in age- and IQ- matched male and female adults with high-functioning ASC. We have documented important similarities and differences between the sexes. In terms of similarities, male and female adults with ASC showed comparable severity of their childhood autistic symptoms, although females self-reported more autistic traits in adulthood. In keeping with one prediction of the extreme male brain (EMB) theory, we found an absence of typical sex differences in ASC in empathizing and systemizing, and in mentalizing performance. Up to 70% of participants also fell into clinically significant ranges on co-morbid psychopathology, a finding of importance in terms of clinical management. However, both males and females had similar levels of current co-occurring anxiety, depression, and obsessive-compulsive symptoms.

In terms of differences between sexes, females presented *fewer* current socio-communication symptoms on the ADOS and had *more* lifetime sensory issues. Within females, there was also a marked difference in performance IQ in those with and without a history of language delay. This pattern of difference as a function of history of language delay was completely absent in males with ASC.

### Similarities and differences in autistic presentation

The first important finding is the strong evidence showing current behavioral sex differences as measured by the ADOS. To demonstrate the importance of this marked difference, it is crucial to point out that the male and female cohorts – matched on age, verbal, performance and full scale IQ – were not different on childhood (“most severe”) core autistic symptom severity measured by the ADI-R. This implies that the two groups were “equally autistic” as children. Therefore, able adult females with ASC compared to males with ASC may achieve more progress in compensatory socio-communication ability. This may be one reason for the more marked sex difference in prevalence of ASC as the behavioral phenotype becomes milder.

One question is whether these women were true cases of ASC. Simply judging from their ADOS scores, only 6 of the 29 (20.7%) females were classified as “autism spectrum”, in comparison to 19 out of the 33 (57.6%) males. However, all these females were diagnosed by experienced clinicians using DSM-IV or ICD-10 criteria, and equally importantly, they scored above cut-off on the ADI-R. Moreover, they scored just as poorly as the males with ASC on high level mentalizing Eyes Test (male mean score 22.3, SD 5.8; female mean score 22.7, SD 6.6). These performances are comparable to a previous independent sample of adults with ASC with similar age and IQ (mean score 21.9, SD 6.6) and are also well below the average observed in the general population (mean score 26.2, SD 3.6) and in above-average IQ controls (mean 30.9, SD 3.0) [58]. Furthermore, these females showed the same empathizing-systemizing profile as their male counterparts. This profile is characterized as the conjunction of low empathy and high systemizing, rendering them “type S” or “extreme type S” cognitive style [29], [32]. Lastly, these females reported an even higher level of autistic traits than males and their scores were well within the range that most people with ASC typically report [31]. All these lines of evidence support the idea that these females were not only diagnostically, neuropsychologically, and cognitively on the autism spectrum (i.e., similar to the males with ASC in cognitive abilities and styles), but were also similar to their male counterparts in terms of childhood symptom severity on the ADI-R.

While this study has documented that adult women with ASC present fewer current socio-communication symptoms, it is an open question as to the underlying reasons for such an effect. Our cross-sectional design is not able to address this question directly and a longitudinal study would be needed to mark developmental changes to explain such differences. However, the contrast between evident childhood symptoms and reduced current autistic interpersonal features fits with anecdotal reports from women on the autistic spectrum [53], [54], [55], [66] as well as our participants' and their caregivers' subjective experiences described in the research interviews. This suggests that able women with ASC may be more motivated and may put more effort into developing compensatory skills that help them to appear “socially typical”. Hence, females with ASC may show different developmental trajectories compared to their male counterparts.

Indeed, experienced clinicians have observed that one reason females (girls or women) with ASC may be less easily identified is because of their ability to “camouflage” their autism [15], [16]. This type of camouflaging may involve conscious, observational learning of how to act in a social setting and by adopting social roles and following social scripts [66]. Hence, a female teenager or adult with ASC may be able to develop reciprocal conversation, social use of affect, gestures and eye gaze, that would place them under the radar for the more commonly understood and recognizable (male) phenotype of ASC [15], [19]. Some of the women with ASC reported they consciously “cloned” themselves on a popular girl in their class whilst at school, imitating their conversational style, intonation, movements, dress-style, interests, and other mannerisms, in minute detail. This suggests that – with the right motivation – learning can be a very effective compensation strategy and could even be exploited therapeutically. Women who adopt these camouflaging strategies nevertheless report that underneath their superficially sociable behavior they are often experiencing high levels of stress and anxiety as they have to work hard to keep up the mask, and that it is exhausting by the end of the day.

Another suggestion is that females with ASC tend to have special interests that are less eccentric or peculiar than their male counterparts [15], [18], [19], [42], or may simply have fewer stereotyped activities [40], [41]. Given the relative insensitivity of ADOS module 4 in picking up such behaviors we could not confidently confirm this possibility. However, the effect of less ADI-R RSB symptom severity in females (though not surviving correction for multiple comparisons) and our qualitative impression from interviews with caregivers was that this may be true for their behavioral presentation in childhood. From a phenotypic standpoint this is an interesting possibility and should be addressed in future research with larger samples across various ages.

Another interesting difference between females and males with ASC were the increased sensory issues in females. Although in DSM-IV sensory issues are not explicitly included in the diagnostic criteria, they are now listed as one of the key symptoms in the proposals for DSM-5 as “unusual sensory behaviors” [38]. This inclusion in DSM-5 mirrors the evidence that both under- and over-responsivity to sensory stimuli may have been an overlooked feature of autism in the past [67]. Indeed, the idiosyncratic sensory and perceptual characteristics of ASC have led to hypotheses about difficulties in multisensory integration [68], enhanced perceptual functioning [69], and the “intense world hypothesis of autism” [70]. More studies are needed to clarify the significance of sensory issues in ASC and its relevance to possible sex differences within ASC. One potential limitation to the observation here is that the ADI-R was not designed to be specifically sensitive to detect sensory symptoms (there are only three sensory items on the ADI-R) and only provides summary information on positive (“unusual sensory interests”) and negative (“undue general sensitivity to noise” and “abnormal, idiosyncratic, negative response to specific sensory stimuli”) sensory issues. Therefore, these findings should be considered preliminary.

An unexpected result that warrants further attention is the more pronounced self-reported autistic traits, as measured by the AQ, in adult females with ASC. Along with the observation of fewer current symptoms on the ADOS, these results suggest that in adulthood, females *show* fewer, but *perceive* more autistic features than males. One possible explanation for this may be that females with ASC are better at masking their autistic features, perhaps because of better self-awareness and self-referential cognitive abilities. Self-referential and social-cognitive traits are related to each other in autism [71], such that increases in one relates to increases in the other. Given the fewer current autistic socio-communication symptoms in females it is possible that this is indicative of some enhanced self-referential ability relative to their male counterparts. Further work testing for differences between males and females in self-referential cognition at the behavioral and neural levels [72] is needed. An alternative explanation could be that, unlike the ADOS which is a state measure of autistic symptomatology that can be influenced by factors such as anxiety during the interview, the AQ is a lifetime questionnaire. The AQ includes not only the state of current functioning but a generalized perception of one's own behavior across the lifespan. It is possible that the adult females with ASC are less socially anxious during the ADOS, which will manifest in lower ADOS scores, but in fact have more autistic characteristics overall.

### Validity of the ADOS for adults with ASC

There are several caveats in interpreting the current set of results. First, we need to consider the validity of the instruments used in this study. Although module 4 of the ADOS was originally designed to assess verbally fluent adolescents and adults, it may not be sensitive enough when used with adults in the average and above-average intelligence range who can camouflage their autistic characteristics. If an individual has learned reciprocal conversation and to use gestures, eye contact and facial expressions in social interaction adequately and frequently, s/he is unlikely to score highly on the ADOS. Yet this does not rule out the existence of other autistic features. Recent attempts to revise the ADOS diagnostic algorithm to improve validity [73] and to create standardized ADOS scores [74] have excluded module 4 due to the possibly distinct behavioral phenotype of adults with ASC. Furthermore, in the original psychometric study of the ADOS [46], in module 4, only 2 out of 16 in the “autism” and 3 out of 14 in the “PDD-NOS” groups were female. In a recent validity study, although ADOS module 4 was able to discriminate ASC from psychopath and typical controls, the results were derived from male adults only [75]. These suggest rather weak evidence to support the same use of the ADOS module 4 for female adults with ASC as a tool for diagnosis. We would suggest that some tell-tale signs among females with good camouflage include speaking and/or writing too much (i.e., a pragmatics deficit), or difficulties with switching attention (e.g. talking to someone whilst composing a text message on a cell-phone). These tell-tale signs, however, warrant further testing. Researchers should use care when interpreting the results of the ADOS in assessing high-functioning adults with ASC. More research is needed to address this validity issue.

On the other hand, whilst this limitation may affect the validity of making a diagnostic judgment for ASC, it does not affect the validity of describing interactive behaviors. A sex difference in ADOS score may not be informative about their underlying diagnostic status, but can still be valid in describing behaviors to certain extent. In this sense, what we observed in terms of immediate interpersonal interaction can be viewed as valid descriptions and comparisons.

### History of language delay

The statistical interaction between history of language delay and sex on performance IQ is also noteworthy. We found that ASC female adults with a history of language delay have significantly lower performance IQ, but only marginally (non-significantly) lower verbal IQ, compared to those without this history. Interestingly this pattern was not observed in males (Figure 1). Although preliminary due to the small sample size of ASC females with language delay (N = 7), it raises an interesting question regarding the role of history of language delay in the development of females with ASC. On average, typical females tend to show more advanced early language development compared to males, but such a difference normalizes later in middle childhood and adolescence [76]. Therefore, a delay in language development in females with ASC may signify more severe deviance or pathology because it carries over to affect nonverbal aspects of cognition. This explanatory mechanism awaits future research.

### Co-occurring psychiatric symptoms

Up to 70% of these adults with ASC scored in the clinically significant range on measures of anxiety, depression, and obsessive-compulsive symptoms. However, both males and females with ASC reported comparable levels on all three measures. Obsessive-compulsive symptoms are phenomenologically related to the RSB domain of ASC and there are also reports suggesting increased obsessive-compulsive symptoms in ASC compared to typical adolescents [61] and adults [62]. Anxiety and depression were the most prevalent axis-1 psychiatric comorbidity in an independent study of adults with ASC [59]. Clinically, close attention to these co-occurring psychiatric symptoms in both males and females with ASC is therefore essential. Our initial look at how these might differ in males and females suggests there is no difference in the presentation of these comorbid psychopathological traits. However, we did not include any physiological state measures related to these dimensions, which might still be different between the sexes.

### Limitations and generalization to other subgroups

Because this is the first study to compare male and female adults with average IQ and ASC, it requires independent replication. Furthermore, given the substantial heterogeneity within ASC [77], [78], our focus on high-functioning adults, and the conservative sample selection procedure (only those reached ADI-R cut-offs were included), one caveat is whether the results from this subgroup of adults will generalize to other subgroups such as younger individuals, those with lower IQ, those with co-occurring medical disorders or commonly associated psychiatric conditions (e.g. fragile×syndrome, epilepsy, ADHD, Tourette's syndrome), or those who have mild autistic features. Finally, participants in this study were recruited mainly from volunteer database and support groups, who are enthusiastic in helping autism research and in facilitating neuroscientists and clinicians' understanding to ASC. They are, however, not fully representative of the whole ASC community.

The present study was set to answer the question “What are the behavioral sex differences and similarities *within* people with ASC?” Thus, the current study is limited in terms of the specificity in making inferences with respect to various types of non-ASC comparison groups. This type of comparison with non-ASC groups is exciting future work that can elucidate the main effects of sex, diagnosis, and interaction between sex and diagnosis. However, while this is an interesting future direction that we are currently investigating, the present inferences about *within*-ASC similarities and differences between the sexes provide valuable information for a more fine-grained phenotypic comparison of male and female adults with ASC.

### Practical implications

High-functioning male and female adults with ASC present somewhat differently in aspects of the behavioral phenotype. Although further studies are necessary to describe the core *common* and *sex-specific* features in the two sexes, practically, the implications to clinicians might be that diagnosis or phenotypic characterization for adults assessed for possible ASC should include not only *direct interview and observation*, but also the collection of *childhood behaviors*, *self-reports* and *neuropsychological assessments*. Judgments made only from immediate interactions might be biased due to camouflaging that may be especially pronounced in females. On the other hand, further understanding may be gained by exploring an individual's coping mechanisms in their everyday social life. In our clinic for adults with suspected ASC, women often only reveal their difficulties in current social functioning via self-report, rather than this being immediately apparent from observation, underlining the importance of an interview with the client about her experiences and perceived difficulties, not just with an informant/parent who knew them when they were young.

Although the present design does not provide direct tests among the competing hypotheses about sex differences in terms of neurobiological and developmental mechanisms in ASC, the findings shed light on females' differential presentation and developmental (compensatory) mechanisms from males, and serve as a basis for future studies. We hope the reported similarities and differences between sexes will contribute to the ongoing debates on the revision of diagnostic criteria for mental health conditions (i.e., DSM-5 and ICD-11), especially in relation to the need for better identification of females on the spectrum [79].
